# Pyrethroids: How They Affect Human and Animal Health?

**DOI:** 10.3390/medicina56110582

**Published:** 2020-10-30

**Authors:** Iga Hołyńska-Iwan, Karolina Szewczyk-Golec

**Affiliations:** 1 Laboratory of Electrophysiology of Epithelial Tissue and Skin, Department of Pathobiochemistry and Clinical Chemistry, Faculty of Pharmacy, Ludwik Rydygier Collegium Medicum in Bydgoszcz, Nicolaus Copernicus University in Torun, 87-100 Torun, Poland; 2 Department of Medical Biology and Biochemistry, Faculty of Medicine, Ludwik Rydygier Collegium Medicum in Bydgoszcz, Nicolaus Copernicus University in Torun, 87-100 Torun, Poland; karosz@cm.umk.pl

**Keywords:** cypermethrin, deltamethrin, permethrin, pathomechanism, health

## Abstract

Pyrethroids are pesticides commonly used in crop protection; in the forestry, wood, and textile industries; as well as in medicine and veterinary medicine to treat parasitic crustacean infestations. They have been found to be relatively safe for humans and animals. Pyrethroids are recommended for personal protection against malaria and virus Zika by the World Health Organization. Pyrethroids act on voltage-gated sodium channels, which cause an influx of sodium ions into the nerve cells and permanent depolarization. They also influence activities of enzymes, especially in nerve and liver cells. Contact of pyrethroids with the skin, digestive tract, and respiratory tract results in their penetration into the body. Due to the importance of the subject, a summary of the current state of knowledge on the toxic effects of pyrethroids was presented in the comprehensive review by Chrustek et al, published in journal *Medicina.* Particular attention was paid to nephrotoxic, hepatotoxic, cardiotoxic, immunotoxic, neurotoxic, and behavioral effects of pyrethroids on human and animal bodies. It could be added that pyrethroids generate oxidative stress, which modifies DNA, RNA, protein, lipid and carbohydrate molecules. However, public awareness of the possible negative effects of the use of insecticides is still low. Further research should be carried out to clarify the molecular basis of the pathomechanism of pyrethroid detrimental action. Proper dissemination of the results seems to be of first importance for public health.

The interest in protecting humans and animals against insects and diseases transmitted by insects, as well as crops against pests, has been around for nearly 200 years. Undoubtedly, due to the growing population of people, farm animals, and growing agricultural areas, the use of pesticides is constantly increasing [1,2,3,4]. Among them, pyrethroids have been considered harmless to humans and animals. The use of first pyrethroids as insecticides began after 1945 [3,4,5]. Nowadays, pyrethroids are widely used in crop protection; in the forestry, wood, and textile industries; in medicine and veterinary medicine to treat infestations of parasitic crustaceans [1,6,7,8,9,10]. They are also used in personal protection against insects, in the form of soaked mosquito nets, sprays, or gels [6,7,8,11,12,13]. Importantly, pyrethroids are used as a preventive measure against the spread of mosquitoes, which is recommended by the World Health Organization (WHO) as a strategy to combat malaria and the Zika virus [9,14,15,16]. For years, anti-malaria programs have been conducted recommending the use of mosquito nets impregnated with deltamethrin and/or permethrin, which belong to pyrethroids [9,10,13]. At present, WHO recommends mosquito nets impregnated with permethrin for personal protection as a part of the prevention of Zika virus infection [10,11].

Historically, pyrethroids are a group of compounds of natural origin isolated from the flowers of the plant *Tanacetum cinerariaefolium* (former name *Chrysanthemum cinerariaefolium)* [1,3,4,5]. Natural pyrethroids are unstable compounds that quickly decompose under the influence of light, therefore the synthesis of derivatives more resistant to radiation, which are also more toxic to insects, has been developed [4,5]. Chemically, pyrethroids are esters of chrysanthemic acid (ethyl 2,2-dimethyl-3-(1-isobutenyl)cyclopropane-1-carboxylate) [4,5]. They occur in two chiral systems—cis and trans—of which cis stereoisomers are characterized by greater activity [3,4]. Permethrin, deltamethrin, and cypermethrin are the most commonly used pyrethroids. A mixture of cis and trans optical stereoisomers of permethrin (1:3, 3-phenoxyphenyl)-methyl] 3-(2,2-dichloroethenyl)cyclopropane-1-carboxylate) is used in insect control agents [15] (WHO permethrin). Permethrin is a chlorinated derivative of chrysanthemic acid, whereas a bromine substituent is unique to deltamethrin ([(S)-cyano-(3-phenoxyphenyl)-methyl](1R,3R)-3-(2,2-dibromoethenyl)-2,2-dimethylcyclopropane1-carboxylate) [14] (WHO deltamethrin). Reagents with cypermethrin ([(S)-alpha-cyano-(3-phenoxyphenyl)-methyl](1R,3R)-3-(2,2-dichlorovinyl)-2,2-dimethylcyclopropane-1-carboxylate and [(R)-alpha-cyano-(3-phenoxyphenyl)-methyl] (1S,3S)-3-(2,2-dichlorovinyl)-2,2-dimethylcyclopropane-1-carboxylate) are a racemic mixture of its optical isomers [16] (WHO cypermethrin). Pyrethroids have been classified by WHO as the fourth group of insecticides [13].

Pyrethroids act on voltage-gated sodium channels [3,4]. Binding of the pyrethroid molecule to the α subunit of the channel causes its permanent opening and prevents it from closing [4]. As a result, the influx of sodium ions into the nerve cells and permanent depolarization occurs [3]. Moreover, pyrethroids reduce the enzymatic activity of acetylcholinesterase (EC 3.1.1.7), modifying the active binding site of the substrate [5]. They also modify the activity of the cytochrome p450 system in brain neurons and in the liver [5]. Pyrethroids are more toxic to insects than to mammals and birds due to the more sensitive sodium channels in the insect nervous system and their lower body temperature [3].

Pyrethroids are lipid soluble, so any contact with the skin, digestive tract, and respiratory tract results in their penetration into the body [1,8,11,17,18,19,20]. The degree of penetration is influenced by the permeability of the barrier [18,19]. It has been proven that a 15-s contact of deltamethrin with the intact rabbit skin is enough to induce a depolarization reaction caused by the influx of sodium ions into the cells [17].

Children and pregnant women are at risk of faster pyrethroid penetration into the body [12,21,22,23,24]. It has been proven that pyrethroids and their metabolites can be found in human milk [12,21], which poses a risk to newborns [12,24].

Pyrethroids were divided into two groups, depending on the type of intoxication symptoms that appeared in a vertebral organism after their administration [3]. Type I pyrethroids, including permethrin, cause the symptoms known as the tremor type syndrome (T), which is characterized by tremors throughout the body, hypersensitivity, aggressive behavior, and ataxia. Type II pyrethroids, represented by deltamethrin and cypermethrin, are associated with the choreoathetosis-salivation syndrome, in which salivation and muscle dysfunction occur [1,3,4,5].

Particularly noteworthy is the fact that it has been proven that the level of pyrethroids in the body, as well as the severity of symptoms of poisoning, depends on the type of diet [1,2,3]. Higher concentrations of pyrethroid metabolites in urine were reported in people on a plant-based diet [2]. However, it should be emphasized that despite the proven negative impact of pyrethroids on human health, at the present stage of knowledge, it is not possible to propose a safer measure for personal protection against insects [14,15,16].

Studies concerning the negative effects of pyrethroids on humans and animals have been conducted for years. The review “Current Research on the Safety of Pyrethroids Used as Insecticides” published in Medicina Lithuania in 2018 [1] is the most comprehensive and extensive summary of the research to date. Particular attention was paid to nephrotoxic, hepatotoxic [25], cardiotoxic [26,27], immunotoxic [28], neurotoxic [29,30], and behavioral [22] effects of pyrethroids and known consequences of their impact on the reproductive system [31] and fetal development [32].

Current knowledge about the site of action of pyrethroids, toxic doses, and induced tissue and organ effects were presented [1]. What deserves special attention are the tables, which collect data on the amount of pyrethroids used in personal protective equipment, metabolites formed after contact with the compound, the main routes of pyrethroid entry into the body, as well as pathomechanisms of negative action and symptoms accompanying poisoning [1].

The article [1] did not address the issues related to the generation of oxidative/nitrosative stress by pyrethroids, associated with the accumulation of oxygen and nitrogen free radicals. The influence of oxidative stress generated by pyrethroids on the modification of DNA, RNA, proteins, lipids, and carbohydrates, both in cells and extracellularly, has been proven in numerous studies [25,33,34,35]. Permethrin and deltamethrin administered to rats have been found to increase the activities of SODs (EC1.15.1.1, superoxide dismutases), GSTs (EC 2.5.1.18; glutathione S-transferases), and CAT (EC 1.11.1.6, catalase) and decrease the activity of GPxs (EC 1.11.1.9, glutathione peroxidases) and the levels of reduced glutathione (GSH) and interleukins 1β, 2, and 13 in the blood plasma [25,33]. Cypermethrin has been reported to increase the concentration of GSH and malonyldialdehyde (MDA), a marker of the lipid peroxidation process, and decrease the activities of CAT and SODs. Additionally, this pyrethroid has shown the ability to accumulate in cell membranes and modify the conformation of transport proteins [34,35].

Nevertheless, public awareness of the possible negative effects of the use of insecticides is still low and further research should be carried out to clarify the molecular basis of the pathomechanisms of the pyrethroid detrimental action. The results of these studies should be widely disseminated. The review [1] presents current scientific data on the impact of three most commonly used pyrethroids, namely, cypermethrin, deltamethrin, and permethrin, on vertebrate organisms, and due to its versatility and topicality it can be recommended as a source of reliable and credible knowledge about pyrethroids.
